# Central Role of the EGF Receptor in Neurometabolic Aging

**DOI:** 10.1155/2012/739428

**Published:** 2012-06-17

**Authors:** Sana Siddiqui, Meng Fang, Bin Ni, Daoyuan Lu, Bronwen Martin, Stuart Maudsley

**Affiliations:** ^1^Receptor Pharmacology Unit, National Institute on Aging, Baltimore, MD 21224, USA; ^2^Metabolism Unit, National Institute on Aging, Baltimore, MD 21224, USA

## Abstract

A strong connection between neuronal and metabolic health has been revealed in recent years. It appears that both normal and pathophysiological aging, as well as neurodegenerative disorders, are all profoundly influenced by this “neurometabolic” interface, that is, communication between the brain and metabolic organs. An important aspect of this “neurometabolic” axis that needs to be investigated involves an elucidation of molecular factors that knit these two functional signaling domains, neuronal and metabolic, together. This paper attempts to identify and discuss a potential keystone signaling factor in this “neurometabolic” axis, that is, the epidermal growth factor receptor (EGFR). The EGFR has been previously demonstrated to act as a signaling nexus for many ligand signaling modalities and cellular stressors, for example, radiation and oxidative radicals, linked to aging and degeneration. The EGFR is expressed in a wide variety of cells/tissues that pertain to the coordinated regulation of neurometabolic activity. EGFR signaling has been highlighted directly or indirectly in a spectrum of neurometabolic conditions, for example, metabolic syndrome, diabetes, Alzheimer's disease, cancer, and cardiorespiratory function. Understanding the positioning of the EGFR within the neurometabolic domain will enhance our appreciation of the ability of this receptor system to underpin highly complex physiological paradigms such as aging and neurodegeneration.

## 1. Introduction

The epidermal growth factor receptor (EGFR) is a 170-kDa single-pass transmembrane (TM) protein. The EGFR belongs to a family of four members: erbB-1/EGFR, erbB2 (HER2/c-neu), erbB3 (Her3), and erbB4 (Her4). The basic EGFR structure contains an immunoglobulin-like extracellular ligand binding domain and an intrinsic tyrosine (Tyr) kinase activity contained within its intracellular carboxyl terminal domain. Extracellular stimulating ligands, for example, epidermal growth factor (EGF), bind to an individual TM receptor and induce a conformational alteration that causes dimerization with another ligand-bound EGFR or with another erbB receptor. The ligand-induced conformational changes then activate the intrinsic tyrosine kinase domains causing subsequent autophosphorylation or transphosphorylation of the opposing receptor in the dimer [1, 2]. The creation of phosphorylated tyrosines then serves to create a dynamic scaffolding domain for downstream signaling molecules that possess SH2 or PTB domains, for example, Grb2 or Shc. Not only are positive signaling molecules recruited in this manner but also factors involved in EGFR internalization, such as Eps15 [3], and eventual lysosomal degradation factors, such as c-Cbl [4, 5]. Many of the signaling functions of the EGFR are mirrored by other members of the receptor tyrosine kinase (RTK) receptor class, for example, ligand-induced tyrosine kinase activation, protein scaffold assembly, and interaction with multiple common downstream factors (phospholipases, phosphoinositide kinases, non-RTKs) [6]. The commonality of function between RTKs indicates that these receptors are highly likely to form important, and strongly inter-connected, links between a diverse range of physiological or pathophysiological activities. 

### 1.1. EGFR Activation Profile Diversity

The activation process of the EGFR typically follows the generic process of EGFR ligand stimulation, tyrosine kinase activation, dimerization and tyrosine (Tyr) phosphorylation, and then signaling protein complex assembly. However, there are considerable functional nuances within this process that serve to profoundly condition and add “*texture*” to the signaling output from these important receptors. For example, the specific nature of the carboxyl terminal domain phosphorylation events provides a mechanism by which a greater variety of signaling information can be conveyed to the intracellular *milieu*. Tyr phosphorylation for EGFR may occur at various sites including Tyr 845, 992, 1045, 1068, 1148, 1173, or 1086. Phosphorylated Tyr residues act as binding sites for proteins containing Src-homology 2 domains (SH2) such as Grb2, Shc and phospholipase C-gamma (PLC-*γ*).Phosphorylation at Tyr 845 in the kinase domain is involved in stabilizing the activation loop, maintaining the active state enzyme, and providing a binding surface for substrate proteins [7, 8]. The non-RTK c-Src is also involved in EGFR phosphorylation at Tyr 845 [9]. The SH2 domain of PLC-*γ* binds at phospho-Tyr 992, which results in activation of PLC-*γ*-mediated downstream signaling [10]. A pair of phosphorylated EGFR residues (Tyr 1148 and Tyr 1173) provides a docking site for the Shc scaffold protein, and both sites are involved in mitogen activated protein kinase (MAPK) signaling activation [1]. Phosphorylation of EGFR at Tyr 1045 creates a major docking site for the adapter protein c-Cbl, leading to receptor ubiquitination and degradation following EGFR activation [4, 5]. The multifunctional Grb2 adapter protein binds the EGFR at phospho-Tyr1068 once the EGFR is activated [11]. Phosphorylation of the EGFR at specific serine (Ser) and threonine residues inhibits EGFR kinase activity. EGFR carboxy-terminal residues Ser1046 and Ser1047 are phosphorylated by CaM kinase II; mutation of either of these serines results in upregulated EGFR tyrosine autophosphorylation [12]. Therefore the pattern and timing of such EGFR posttranslational phosphorylation events strongly affect its cellular disposition and eventual signaling capacity. Such a wide flexibility of function is typically characteristic of protein factors that are likely to form multidimensional interactions within more complex physiological paradigms [13, 14].

### 1.2. Diversity of EGFR-Stimulatory Factors

The EGFR possesses a highly complex relationship with its stimulating ligands/factors. Endogenous peptide-based ligands that can activate the EGFR include epidermal growth factor (EGF), transforming growth factor (TGF)-*α*, amphiregulin (AR), heparin-binding EGF (HB-EGF), betacellulin, epiregulin (ER), and epigen [15]. The EGFR is almost unique amongst receptor systems in that it serves as a molecular integration site for multiple types of stimuli including: peptide ligands, metal ions, ultraviolet and gamma radiation, osmotic shock, membrane depolarization, and oxidative radicals [16]. This wide range of stimuli again reinforces the concept that the EGFR acts as a functional keystone in “higher-order” complex systems that may underpin multifactorial global somatic actions such as aging and metabolism.

The EGFR can be activated by small transition metal ions such as zinc and copper (Cu) [17]. In addition to simple metal ions, ultraviolet (UV) radiation activates the EGFR by two ligand-binding independent mechanisms: Tyr phosphorylation of the receptor [18–20], and via oxidative inhibition of the RPTP-*κ* [21]. It appears that the generation of reactive oxygen species (ROS) may in some cases be secondary to EGFR activation. These ROS may lead to the reversible inactivation of a crucial protein for EGFR activation, that is, protein tyrosine phosphatases (PTPs) by oxidizing the catalytic cysteine in their active site [22, 23].

Heptahelical G-protein-coupled receptors (GPCRs), which classically were not primarily associated with tyrosine kinase signaling cascades, have subsequently been demonstrated to exert a profound regulatory capacity over RTK systems, including the EGFR [24–29]. This GPCR-RTK regulatory capacity has been demonstrated for multiple types of GPCRs, for example, muscarinic, bombesin, thrombin, endothelin, lysophosphatidic acid (LPA) receptors, which perhaps suggests that this is a common function for GPCRs in most physiological processes. There appear to be multiple mechanisms that can mediate this functional GPCR-RTK interaction, however one of the most studied involves the GPCR-induced processing of pro-HB-EGF to its soluble form through the activation of intramembrane metalloproteinases [30]. Matrix metalloproteinases (MMPs) shed the pro-form of the EGFR ligands which are then locally liberated and able to induce classical EGFR activation. It is interesting to note that this so-called G protein-coupled receptor RTK “transactivation” is a process not only limited to heptahelical GPCRs. For example, the insulin-like growth factor-1 receptor (IGF-1R), classically considered to possess an RTK structure, is actually a “*functional G protein-coupled receptor*” as it is able to stimulate guanine nucleotide exchange event within its associated G proteins [31]. IGF-1-mediated stimulation of the IGF-1R can therefore result in a “transactivation” of the EGFR in a manner similar to that induced by heptahelical GPCR activity [32]. This interaction between the IGF-1R and the EGFR perhaps forms one of the first points of molecular integration between neurological and metabolic activities. In addition to this functional system bridge between neuronal and metabolic systems, ligands of G*α*q/11-coupled GPCRs that are associated with insulin resistance, such as serotonin, endothelin-1, and thrombin, may also stimulate HB-EGF production and transactivate EGFR in 3T3-L1 adipocytes. Serotonin has been shown to additionally have this effect in primary adipocytes and myotubes [33]. Not only can serotonin induce activation of the EGFR but it also appears to be able to regulate the posttranslational activity, via serine phosphorylation and the mTOR pathway, of the insulin/IGF-1 receptor-associated protein, IRS-1 [33]. IRS-1 and -2 are insulin receptor-associated scaffold proteins essential for effective glucose metabolism in multiple energy-regulatory tissues such as the liver [34]. This serotoninergic synergy between IRS-1 phosphorylation and HB-EGF-mediated EGFR stimulation has been demonstrated to play a key role in serotonin-induced insulin resistance [35]. This molecular linkage may underpin the ability of antiserotoninergic therapy amelioration of glucose tolerance in diabetics [36]. Insulin resistance has been shown to have causal relationships with proinflammatory cytokines (including adipokines) [37], prolonged/amplified insulin stimulation, oxidative stress, and endoplasmic reticulum stress [38–40]. Indicating the significant interaction of the EGFR system with other signaling modalities, it has been shown that activation of the growth hormone cytokine receptor can induce phosphorylation of EGFR in preadipocytic fibroblast cell lines. This cytokine receptor-mediated transactivation event effectively modulates EGF-induced EGFR trafficking and signaling [41]. It is important to note that the growth hormone ligand and receptor system is one of the most important factors that controls both IGF-1 expression and secretion as well as somatic energy metabolism.

It has been revealed in recent years that the Toll-like receptor (TLR) system is important for neurological health [42]. Ligand activation of multiple TLR isoforms has been demonstrated to induce subsequent activation of EGFRs [43, 44]. This pathway involves the activation of nicotinamide adenine dinucleotide phosphate and the generation of reactive oxidative species (ROS). Subsequent activation of tumor necrosis factor (TNF)-*α*-converting enzyme (TACE) leads to the liberation of TGF-*α* from the epithelium and productive ligation of the EGFR [45]. TLR2 activation by lipoteichoic acid leads to a disintegrin and metalloproteinase (ADAM)-10-induced cleavage of HB-EGF, another EGFR ligand. Amphiregulin has also been shown to be released in response to TNF-*α* [46].

It is clear therefore that in addition to its widespread expression and highly textured signaling activity, the EGFR is subject to multiple and highly diverse signaling inputs both from other receptor systems and small molecules and ions. These combined molecular factors and emerging evidence of functional overlap of EGFR activities from neuronal to metabolic systems suggest that the EGFR system may exert a “near omnipotent” function in aging biology.

## 2. EGFR and the Control of Neurometabolic Physiology

The EGFR, and its axis of ligands demonstrate a widespread expression profile across the central nervous system (CNS) [47]. Substantial EGFR expression is found in the neocortex and limbic cortex, cerebellum, cerebrovascular endothelial cells [48, 49], and the midbrain [50]. EGFR has been detected in the hippocampal pyramidal cells, Purkinje cells, large multipolar neurons of the dentate nucleus, anterior horn cells, dorsal root ganglion cells, cells of the dorsal nucleus of Clark, intermediolateral column cells, and ependymal cells [51]. In the human fetal brain, EGFR has been shown to be expressed in the subventricular zone (SVZ) [52], hippocampus, and cerebellum [53]. This expression pattern in the two primary areas of adult neurogenesis suggests that the EGFR could also play a strong role in age-related neuronal survival and regeneration. The EGFR has also been implicated in chemotactic migration in the developing telencephalon with implication of HB-EGF involvement [54]. EGF appears to act as a mitogen for neural stem and progenitor cells (NS/NPCs) in the CNS as well as numerous other cell types involved in neurometabolic activity. Along with EGF, HB-EGF and TGF-*α* also promote proliferation in the SVZ of an adult mouse HB-EGF [55]. *In vitro*, EGF maintains NS/NPCs in the proliferative state, whereas in the normal rodent brain, it induces proliferation and migration in the SVZ. EGF can also increase neuronal replacement in the ischemia-injured adult striatum. In addition, Sun et al. (2010) found that EGF is neuroprotective rather than neurogenic when protecting the brain from injury [56].

Notch proteins are a family of transmembrane receptor proteins with repeated extracellular EGF and titular Notch domains. Notch interacts with cell-bound ligands (*Delta-like*, *Jagged*) that facilitate an inter-cellular signaling pathway that is critical in cellular and tissue development. Notch family members regulate developmental processes by controlling cell fate decisions [57]. Notch and EGFR have fundamental and selective roles in the maintenance of NS/NPCs in the SVZ. Notch signaling promotes proliferative signaling during neurogenesis and its activity is inhibited by *Numb* to promote neural differentiation. The *Numb* gene product controls binary cell fate decisions in peripheral and central nervous systems during neurogenesis. Notch and EGFR pathway interaction regulates neural stem cell number and self-renewal [58]. Altering particular signaling mechanisms in selective cell types of the SVZ can cause profound changes in the overall cell composition of this neurogenic region of the adult brain. Defining interactions and homeostatic mechanisms that occur between different types of SVZ cells under normal conditions provides crucial information on possible alterations of specific signaling pathways that might occur under pathological conditions or after brain injury.

The aspartyl protease, *γ*-secretase, is a multisubunit protease which mediates the coordinated intramembrane proteolysis of both Notch and amyloid-precursor protein (APP), which are both implicated in the etiology of Alzheimer's disease (AD). In some cell types the expression of EGFR and *γ*-secretase have been reported to be inversely related to each other, suggesting the existence of strong functional connection between these two systems [59]. In a study of squamous cell carcinoma, Notch and EGFR were shown to participate in the tumor suppressor function of *γ*-secretase, again reinforcing the existence of a physiologically-relevant EGFR-*γ*-secretase interaction [60].

Given the considerable evidence for a strong role of the EGFR in neural stem cell development it is unsurprising that one of the functional sequelae of this activity is the maintenance of memory pattern formation in the hippocampus. Two of the most important regions of the CNS for adult stem cell-mediated neurogenesis are the hippocampal CA1 and dentate gyrus (DG) [61]. Membrane-tethered pro-ligand, HB-EGF, is found in cells within many regions of the CNS, for example, cerebellum, cerebral cortex as well as the hippocampus [62]. The specific hippocampal expression of HB-EGF and the association of the EGFR with neurodevelopmental processes have led to the implication of EGFR signaling in synaptic plasticity and memory formation [55]. HB-EGF knockout mice demonstrate also reduced CNS expression levels of neurotrophic factors including brain-derived neurotrophic factor (BDNF), which is a ligand for the tropomyosin-receptor kinase- (Trk-) B receptor [55]. Expression levels of BDNF and TrkB receptor activity are both strongly correlated to the regulation of synaptic architecture and the ability to form and retain memory patterns. The hippocampal regions, CA1/DG, demonstrate a tremendously high requirement for energetic support due to their constant stimulation both from sensory inputs and higher areas of the brain. Their huge energetic load therefore makes CA1/DG neurons extremely sensitive to the metabolic status of the individual [63–65].

One of the strongest points of physiological neurometabolic intersection may occur via the neurometabolic control of reproductive activity. It has been demonstrated that there are strong gender-specific molecular mechanisms that link hippocampal cognitive function to centrally-controlled reproductive behavior [66–69]. It has also been demonstrated that the EGFR is localized to the anterior pituitary [70] along with EGF [71] and TGF-*α* [72], implicating them in the reproductive hypothalamic-pituitary axis [73]. In the anterior pituitary, EGFR has been identified in lactosomatotrophs [74], corticotrophs [75], and gonadotrophs [76, 77]. The EGFR has been directly implicated in corticotroph proliferation and hormone secretion, while TGF-*α* has been implicated in EGFR-dependent estrogen-mediated corticotroph cell proliferation [78, 79]. In addition to these reproductive functions, EGFR activity is also closely associated with the dynamic regulation of prolactin transcription and synthesis [80].

## 3. Role of the EGFR in Neurometabolic Pathophysiology

As one would expect from its multifactorial biological activity, the genomic inactivation of EGFR by homologous recombination results in profound systemic effects. EGFR genomic inactivation can induce three different phenotypes that range from peri-implantation lethality to postnatal lethality [81]. EGFR-inactivated mice die at different stages of development depending on their genetic background; EGFR mutant mice may die at gestation (129/Sv), at birth (C57Bl/6), or they may live up to 8 or 20 days (CD1, MF1, 129Sv/J Swiss Black or C3H) [82–86]. After parturition, the surviving animals may suffer from impaired epithelial development in organs such as the skin, lung and gastrointestinal tract [83] as well as the placenta. Furthermore, there is a strain-independent postnatal neurodegeneration in the frontal cortex, olfactory bulb, and thalamus in surviving EGFR-null mice [82]. This neurodegeneration is characterized by massive apoptosis and upregulation of c-*fos* [82]. The mildest form of EGFR inactivation leads to epithelial immaturity and postnatal death due to respiratory failure and necrotizing enterocolitis-like lesions in the intestine [81]. The defects seen in this “postnatal lethality phenotype” manifest in the classical EGF-responsive organs (skin, intestine) and organs undergoing branching morphogenesis during development (lung, kidney, mammary gland, pancreas, and prostate) and, thus, are in concordance with the concept of EGF family members being important epithelial mitogens. Intestinal changes observed in the EGFR-inactivated mice differ in the level of severity, the endpoint being severe mucosal lesions and necroses (cell death) [81].

Within the CNS, it has been suggested that the EGFR is likely to mediate the effects of EGF/TGF-*α* on neuronal differentiation [87], survival [88–90], as well as glial proliferation [91, 92]. While many neurons of the CNS constitutively express the EGFR [48, 49], glia and endothelial cells demonstrate induced receptor expression following acute injury or chronic neurodegeneration [48, 49, 93–95]. Glial cells are vital for the generation of a dynamic support structure for CNS activity in the face of altered energy availability or oxidative stress [96]. TGF-*α*-gene expression in glial cells is a component of the hypothalamic response to injury [97] and it has been reported that during development, the increased bias towards glial differentiation is not dependent on EGFR signaling [98]. EGFR expression appears to the crucial for the proliferation and differentiation of astrocytes [92], however, it is only one of a number of means by which astrocytes can be induced. Burrows et al. (1997) demonstrated that misexpression of EGFRs promoted astrocyte development prematurely *in vivo *[99]. Furthermore, it was suggested that EGFRs may induce the development of astrocytes by regulating the responses of progenitors to various extrinsic signals such as leukemia inhibitory factor (LIF) and bone morphogenetic proteins (BMPs) [99]. It was also later demonstrated that EGFRs elevate STAT3 expression and increase its phosphorylation by LIF and that EGFRs further regulate LIF downstream of STAT3 but they do not regulate changes in responsiveness to BMPs [100].

Mice that do not possess EGFR develop neurodegeneration involving the frontal cortex and olfactory bulbs. It has been shown that EGFR signaling controls cortical degeneration by the regulation of cortical astrocyte apoptosis [101]. Midbrain astrocytes where EGFR is absent are not affected; however, some mutant cortical astrocytes possess an increased incidence of apoptosis which is mediated by an Akt-caspase-dependent mechanism and these cells demonstrate a reduced ability to support neuronal survival [101]. These results suggest two functionally distinct astrocyte populations exist, which are differentially dependent on EGFR signaling for their survival and also for their ability to support neuronal survival [101]. These spatial differences in astrocyte composition provide a mechanism for the region-specific neurodegeneration in EGFR null mice [101].

TGF-*α* contributes to the neuroendocrine regulation of female puberty by stimulating the release of luteinizing hormone-releasing hormone (LHRH) [97]. It has been proposed that TGF-*α* may be involved in a glial-neuronal interaction where it first stimulates PGE2 from glial cells which in turn elicits LHRH from neuronal terminals [102]. Interestingly astrocytes, but not LHRH neurons, express EGFR [103]. Disruption of astroglial EGFR signaling leads to irregular estrous cycles and a decreased secretion of LH in female mice which eventually can cause infertility [104]. The EGFR and TGF-*α* are present in the suprachiasmatic nucleus (SCN), which is the core circadian pacemaker. The SCN releases factors acting locally within the hypothalamus, more specifically in the neurons of the subparaventricular zone. It has also been reported that EGFR has a circadian-time-dependent neuromodulatory function in the SCN [105, 106]. TGF-*α* has been implicated as an inhibitor of locomotion and sleep-wake cycles [107]. In hamsters, these data are confirmed, and, furthermore, it was shown that central administration of TGF-*α* induced weight loss [108].

## 4. EGFR and Neurodegenerative Disease

AD is one of the most prevalent familial and sporadic neurodegenerative disorders. One of the strongest risk factors for AD appears to be advancing age. In addition to the clear neurological basis of AD, the contribution of metabolic factors to this disorder as well as other neurological processes has been the subject of considerable recent scrutiny [65, 109–111]. In AD pathology, aggregates and deposits of the protein fragment *β*-amyloid (plaques) and twisted strands of the protein Tau (neurofibrillary tangles) induce the generation of nerve cell damage and cell death in areas of the brain including the cortex and especially the hippocampus [110, 112–114]. With respect to an association with AD etiology it has been demonstrated that EGFR expression in the rat brain reduces with age both in males and females [115]. AD-related neuritic plaques observed in the cerebral cortex and hippocampus of patients with AD also immunostain positively for EGFR [93]. In addition, it has been demonstrated that EGFR expression is upregulated in astrocytic cells in AD [116] and that EGFR-null astrocytes from mutant cortices in mice have an impaired proliferative capacity both *in vitro* and *in vivo *[82, 86]. With respect to the potential involvement of the EGFR in the etiology neurometabolic pathophysiologies, such as AD, a strong role for metal ion activity is likely. It has been demonstrated that AD is often associated with a central nervous imbalance of transition metal levels which may lead to neurotoxicity. Transition metals facilitate oxygen transport and its eventual usage by nervous tissue. The same metal ion chemistry if dysregulated, for example, Cu imbalance, can induce the generation of reactive oxygen species (ROS) which are strongly associated with AD and pathological aging [117]. Interestingly, clioquinol-Cu metal complexes, which are cell-permeable, can be employed to ameliorate AD pathology via their ability to stimulate the EGFR in neuronal-derived cell lines causing a reduction in *β*-amyloid levels [118]. This metal ion process also appears to involve the activation of TM matrix metalloproteinases (MMPs) as well the nonreceptor tyrosine kinase c-Src [118]. Iron is another metal implicated in AD through its capacity to modulate the ability of amyloid peptides to yield ROS [119] which then may serve to exacerbate the disorder further [120].

EGFR immunoreactivity has been localized to the brain, pituitary, skin vascular endothelial cells of clinically diagnosed, pathologically confirmed AD patients when samples were collected postmortem [95]. It has also been reported that EGFR is localized to the lumenal surface of endothelial cells and that EGFR immunoreactivity in brain vasculature was present in all assessed elderly patients with dementia, when compared to nondemented patients [94]. As microvascular damage and hypertension are strongly associated with intermittent ischemia, hypoxia, and AD-related pathology, it seems likely that the cerebrovascular activity of the EGFR is an important aspect of its role in neurodegenerative pathophysiology. In addition to a potential role in AD, it has been shown that there is an increased expression of EGFRs in the striatum in chronic Parkinsonian syndromes but not in acute models of the disease [121]. Depletion of neuronal dopamine is observed in Parkinson's disease and this subsequently would lead to decreased precursor cell proliferation in the SVZ as well as a reduction in local EGF production. EGFR positive cells have been reported to be depleted in SVZ in Parkinson's disease as well: these data collectively indicate a role of dopamine-EGFR signaling loop in regulation of neurogenesis of dopaminergic neurons [122]. This process appears to involve an Wnt5a-dopamine D2 receptor interaction in association with the EGFR [123]. Taken together there appear to be multiple and mechanistically diverse routes by which the EGFR may be involved in the neuronal aspect of aging-related disorders and neurodegeneration.

## 5. EGFR Regulates Aging-Related Metabolic Activity

Aging is considered to be one of the most complex physiological processes known. Despite its enormous molecular complexity there appear to be several molecular mechanisms in which the aging process can considered in a generic sense, for example, disruption of hormonal axes, accumulated oxidative stress, increasing nucleic acid instability, and a reduction of metabolic efficiency. Even among these common aging factors, metabolism for one appears to be vital for all of the physiological aspects of aging. It is not surprising therefore that many of the effective antiaging strategies intersect with this important factor [109, 124–130]. Therefore, if metabolic function is an overarching factor in the aging process, the question arises as to how the multifunctional EGFR might affect this profoundly important process.

Historically, however, the EGFR has been more commonly associated with cancer biology as opposed to neuronal function, metabolism, or aging. Given that the EGFR is one of the most widely expressed growth factor receptors, it is implicated in playing an important role in the proliferation of a wide variety of cell types in addition to the previously studied cancerous cells [131]. For example, the EGFR is amplified and overexpressed in tumors of epithelial [131] and glial origins [132] as well as other numerous neurometabolic-related tumors observed in humans. Viruses often associated with cancers, such as the hepatitis B virus, which is associated with hepatocellular carcinoma, lead to upregulation of EGFR signaling [133, 134]. Recent research however has started to reveal a more widespread contribution of the EGFR to complex physiological processes such as energy metabolism. In gastric cell lines, transactivation of the EGFR intimately involves leptin-signaling. Leptin is a cytokine hormone secreted by adipocytes that regulates body weight by decreasing food intake and energy expenditure [135]. EGFR is also constitutively expressed in epithelium and airway smooth muscle (ASM) [136] as well as in hepatocytes [137]. The liver represents one of the most important organs with respect to the global somatic control of both energy expenditure and nutrient storage. Interestingly, it has been reported that the EGFR demonstrate a 2-fold higher expression in adult rat males compared to females and this phenomenon may be influenced by secretory rhythm of growth hormone (GH) in the pituitary [138].

Perinatal deletion of EGFR in hepatocytes results in decreased body weight, whereas deletion in the adult liver does not appear to affect body mass. Although liver function was not affected, after partial hepatectomy, mice lacking hepatic EGFR exhibit increased mortality and elevated serum transaminases, indicative of liver damage. EGFR has also been shown to be a critical regulator of hepatocyte proliferation in the initial phases of liver regeneration [139]. Several studies have investigated various regulatory factors that contribute to the affinity of EGF to EGFR in rat hepatocytes, for example, thyroid hormones [140], age [141], sex [141], fasting state [142], liver regeneration [143], and an imposed experimental diabetic state [144–146]. In accordance with this, a decreased hepatic EGFR expression has been reported in diabetic mice [147, 148]. Given the aforementioned molecular connectivity between the EGFR and the insulin/IGF-1/GH signaling systems it is logical that insulin may be one of the most important factors that regulate EGFR gene expression in the liver [147]. EGF and EGFR have been shown to be involved in an antiapoptotic effect in mouse hepatocytes [149] while EGF decreases the glucose transporter 2 expression level in chicken hepatocytes via PKC-MAPK [150]. Insulin deficiency causes a decrease in EGFR and its mRNA gene expression: therapeutic introduction of exogenous insulin restored the EGFR expression to control levels [147]. In addition to the insulinotropic regulation of hepatic EGFR function, the EGFR can be tyrosine-phosphorylated in the liver in a time- and dose-dependent manner in response to GH [151].

Carcinoembryonic antigen-related cell adhesion molecule 1 (CEACAM1) is a cell adhesion protein and recently identified a substrate of the EGFR [152]. Once phosphorylated by EGFR activation, CEACAM1 appears to mediate increases in insulin sensitivity and can decrease insulin-dependent mitogenesis *in vivo*. An attenuated response to insulin stimulation is causally related to obesity, type 2 diabetes, and metabolic syndrome, and improved whole-body insulin sensitivity is important in the treatment of these metabolic disorders [153]. CEACAM1 therefore appears to play a central role in connecting EGFR activity with the control of central obesity/insulin resistance. The connection between EGFR activity and obesity is further reinforced by the observation that obesity is strongly associated with an increased mortality rate in cancer patients [154, 155]. The mechanism(s) linking altered metabolic pathways to mitogenic activity may involve EGFR activation by fatty acids independently of the cognate growth factor ligands of EGFR [156, 157]. Free fatty acids (FFAs) are upregulated in fasting plasma of obese individuals [158]. Rather than being considered a passive organ adipose tissue is now appreciated as a dynamic endocrine regulatory organ. In addition to releasing FFAs [159] HB-EGF can be released from adipose tissue in obese individuals into the portal circulation [160]. Both FFAs and HB-EGF possess the capacity to engender EGFR activation in epithelial cells [152].

As we have discussed, in addition to its strong role in hepatic function, the EGFR also appears to coordinate multiple aspects of the glucose metabolic system. EGFR and the EGFR ligands, especially TGF-*α*, is functionally expressed in the developing pancreas [161]. EGFR expression is essential for normal pancreatic development and important in postnatal *β* cell growth [162]. Furthermore, EGFR signaling is essential for pancreatic islet *β* cell mass expansion during a high-fat diet and pregnancy. In these scenarios cellular replication appears to be the primary mechanism for the compensatory *β* cell mass expansion [163]. EGFR signaling however was not crucial to increase *β* cell proliferation after pancreatic duct ligation, which serves as a model for islet neogenesis. In EGFR-deficient mice, the development of *β* cells occurs at a later stage and the nascent *β* cells have a definite migratory defect [162]. EGFR ligands, EGF, HB-EGF, and betacellulin have all been implicated in *β* cell replication, differentiation and lineage determination of developing islet cells [164–167]. In addition to direct ligand-induced activation of the pancreatic EGFR, local GPCR-mediated transactivation of the receptor also appears to be potentially important in the EGFR-mediated regulation of glucose metabolism [168]. Glucagon-like peptide 1 (GLP-1) is secreted by intestinal L-cells in response to fat meals and carbohydrates [169–171]. In addition to its direct roles in the alimentary canal, for example, regulation of gastric emptying, GLP-1 also can functionally regulate *β* cell function in the pancreas, thereby providing another mechanism to control energy metabolism. The GLP-1 peptide activates its cognate Class B-type GLP-1 receptor to induce a c-Src-dependent EGFR transactivation process. This signaling system involves a proteolytic processing of membrane-anchored betacellulin, or other EGF-like ligands, that results in a strong PI3-kinase activation resulting in an effective increase in *β*-cell proliferation [172].

A common pathological aspect of metabolic aging is a slow inevitable generation of global insulin resistance [13]. Insulin resistance and the associated metabolic syndrome lead to a reduction in the ability of multiple cell types, and especially neurons, to uptake glucose, regulate calcium homeostasis and respond positively to trophic and neurotrophic stimuli [173]. In addition to generating a global disruption of neuronal energy balance, protracted insulin resistance leads to a loss of vascular smooth muscle cell (VSMC) functional regulation and promotion of VSMC migration [174]. The latter is crucial in atherosclerotic development and wound healing. Insulin has also been implicated in epidermal wound healing though EGFR signaling [174]. Insulin can induce transactivation of EGFR by ADAM-mediated HB-EGF-dependent process in VSMCs [174]. In quiescent fibroblasts, the mitogenic effect of EGF requires activation of the IGF-1R by circulating ligands including IGF-I, IGF-II, or insulin [175]. As alterations in VSMC tone and function are likely to affect systemic hypertension, it is interesting to note that elevated plasma glucose can also result in the transactivation of the EGFR in renal disorders [176].

## 6. EGFR and TrkB Signaling: Potential Mediators of the Neurometabolic Interface

Considerable scientific progress has been made in studying EGFR since Cohen and Carpenter's publication (1975) that EGF induces precocious eyelid opening in neonatal mice [177]. The EGFR demonstrates an unprecedented number of functional interactions with other receptors, ligands, and molecules other than its own cognate ligands. Understanding these interactions is crucial to understanding the many different levels at which it is contributing to neurometabolic processes. However, the placing of the EGFR in such a central and important role in aging is overly simplistic. There are of course likely to be other factors that also possess a multidimensional impact upon neurometabolic function. For example, the interaction of the EGFR with the metal ion complexes leads to the resultant generation of downstream signaling phenotypes which are more characteristic of that engendered by the activation of the neurotrophic TrkB signaling system. The activity of the neurotrophic receptor family is already appreciated to be incredibly important to the presence and generation of AD pathology [178]. Trk receptors are necessary for the survival, differentiation and maturation of the developing brain [179]. Multiple lines of evidence have demonstrated a reduction of brain-derived neurotrophic factor (BDNF), the cognate ligand for the TrkB receptor, in the brain of AD patients [180–183]. Furthermore, TrkB has been reported to be important for LTP, a process considered to underpin memory function, in hippocampal CA1 neurons [184, 185]. Both the TrkB and EGFR signaling systems could potentially form a functional link that generates a combined regulatory system deeply associated with the majority of signaling systems controlling the aging process. It is interesting to note that both the TrkB and EGFR receptor systems can be directly linked through GPCR “*transactivation*” signaling mechanisms. Therefore it is likely that a “higher-order” synergy between TrkB and the EGFR could account for a significant component of the neurometabolic aging phenotype. Also through their common GPCR interaction in this “super-system” could be easily targeted in a therapeutic manner as currently over half of the effective pharmacopeia is designed to employ GPCR signaling systems. Such potential agents targeting this EGFR-TrkB network may be able to exert tremendously powerful therapeutic effects in aging and neurodegenerative paradigms.

## 7. Conclusion

In this paper we have attempted to uncover the context of neuronal and metabolic interaction at the molecular level during the aging process. Enormously complex systems such as aging entail functional interaction of a myriad of physiological and molecular signaling systems. To provide a workable molecular framework on which to regulate and control such an enormous system it is likely that the existence of factors that can bridge, and adapt to, a multiplicity of signals, receptors, cell types, and tissues is crucial to the orchestration of the overall network. It is at this level that individual functional entities, such as the EGFR receptor system, may come to prominence. In its ability to interact and regulate multiple neuronal and metabolic functions across lifespan, the EGFR may indeed be one of the most important molecules whose enhanced study could yield future insights of tremendous importance for gerontology. In our paper we have drawn together evidence to aid the appreciation of the truly multidimensional role of EGFR at the systemic level in neurometabolic processes and in the neurodegenerative trajectories seen in the aging process.

## Abbreviations

AD: Alzheimer's disease
ADAM: A disintegrin and metalloproteinase
Akt: Ak-thymoma8
APP: Amyloid-*β* precursor protein
AR: Amphiregulin
ASM: Airway smooth muscle
BDNF: Brain-derived neurotrophic factor
BMPs: Bone morphogenetic proteins
CA1: Cornu Ammonis 1
CaM kinase II: Ca^2+^/calmodulin-dependent protein kinase
c-Cbl: Casitas B-lineage lymphoma protooncogene
c-fos: Fbj osteosarcoma oncogene
c-Src: Rous sarcoma oncogene
CNS: Central nervous system
CQ: Clioquinol
Cu: Copper
DG: Dentate gyrus
EGF: Epidermal growth factor
EGFR: Epidermal growth factor receptor
Eps15: Epidermal growth factor receptor phosphorylation substrate-15
ER: Epiregulin
ERK: Extracellular-signal-regulated kinases
FFAs: Free fatty acids
GH: Growth hormone
GLP-1: Glucagon-like peptide 1
GLP-1R: Glucagon-like peptide 1 receptor
GPCR: G-protein coupled receptor
Grb2: Growth factor receptor-binding protein 2
HB-EGF: Heparin-binding epidermal growth factor
IGF-1: Insulin growth factor-1
IGF-1R: Insulin growth factor-1 receptor
IRS-1: Insulin receptor substrate-1
LHRH: luteinizing hormone-releasing hormone
LIF: Leukemia inhibitory factor
LPA: Lysophosphatidic acid
LTP: Long-term potentiation
MAPK: Mitogen-activated protein kinase
MMPs: Matrix metalloproteinases
NS/NPCs: Neural stem and progenitor cells
PBMCs: Peripheral blood mononuclear cells
PGE2: Prostaglandin E2
PI3K: Phosphoinositide 3 kinase
PKC: Protein kinase C
PLC-*γ*: Phospholipase C gamma
PTB: Protein tyrosine binding domain
p-Tyr: Phosphorylated tyrosine
Ras: Rat sarcoma viral oncogene
RPTP-*κ*: Receptor protein tyrosine phosphatase kappa
ROS: Reactive oxygen species
RTK: Receptor tyrosine kinases
Ser: Serine
SH2: Src homology 2 domain
Shc: Src homology construct
Src: Rous sarcoma protein
STAT3: Signal transducer and activator of transcription 3
SVZ: Subventricular zone
TACE: Tumor necrosis factor alpha-converting enzyme
TGF-*α*: Transforming growth factor alpha
TLR: Toll-like receptors
TM: Transmembrane
TNF-*α*: Tumor necrosis factor alpha
Trk: Tropomyosin receptor kinase
Tyr: Tyrosine
UV: Ultraviolet
VSMC: Vascular smooth muscle cell.
